# Continuity, psychosocial correlates, and outcome of problematic substance use from adolescence to young adulthood in a community sample

**DOI:** 10.1186/1753-2000-1-12

**Published:** 2007-10-11

**Authors:** Hans-Christoph Steinhausen, Susanne Eschmann, Christa Winkler Metzke

**Affiliations:** 1 Department of Child and Adolescent Psychiatry, University of Zurich, Neumuensterallee 9, CH 8032 Zurich, Switzerland

## Abstract

**Background:**

The study of the continuity, psychosocial correlates, and prediction of problematic substance use (PSU) across time from adolescence to young adulthood.

**Methods:**

Substance use was studied in a cohort of N = 593 subjects who had been assessed at three times between adolescence and young adulthood within the Zurich Psychology and Psychopathology Study (ZAPPS). Based on the frequency of tobacco, alcohol, and cannabis consumption, groups with PSU were defined at each of the three measurement points in time and compared to the rest of the sample. Comparisons included questionnaire data regarding emotional and behavioural problems, life events, coping style, self-related cognitions, perceived parenting style, perceived school environment, and size and efficiency of the social network.

**Results:**

The size of the groups with PSU increased continuously across time. The cross-sectional correlates of PSU were characterized by a similar pattern that included higher scores for externalizing behaviour, and both number and negative impact of life events across all three times. At time 1 and 2 subjects with PSU also experienced less favourable parenting styles and school environments. Longitudinally, PSU in young adulthood was predicted most strongly and persistently by previous risk status, externalizing problems and male gender.

**Conclusion:**

Problematic substance use is a major problem in youth. Its contributing pattern of associated and predictive psychosocial variables can be identified in the community.

## Introduction

In a recent review of drug abuse research it has been stated that some of the advancements parallel concepts that are also part of the developmental psychopathology approach. Among various research areas, this perspective includes antecedent and co-occurring psychopathological conditions and other problem behaviour [1]. To date, there is only a small number of longitudinal studies that have taken this perspective by studying the association of adolescent substance use with adult outcome [e.g. [2-5]] or the developmental antecedents, patterns, and correlates of substance use in adolescence [6-10].

Within the developmental perspective of adolescent substance use, various risk factors including life stress, personality features, parental behaviour, peer influences, school characteristics, and other environmental features have been studied as can be delineated from both major reviews and empirical studies [6,11-17]. The frequent co-occurring mental disorders and behaviour problems have been addressed particularly in another series of recent reviews with a strong emphasis on co-morbid conduct disorders or externalizing problems [13,18-20].

The present study attempted to shed some more light on the developmental trajectories of substance use from adolescence to young adulthood. Based on data from a longitudinal study, the main focus was on problematic substance use rather than on substance use disorders. In accordance with various studies, weekly or daily consumption of tobacco and/or alcohol, problem and heavy drinking aiming at drunkenness, and cannabis use more frequently than three times in the past month was considered to reflect problematic substance use [21]. The major aim of the present study was the analysis and predictive power of selected psychosocial correlates across time in subjects with problematic substance use (PSU). The psychosocial correlates of the longitudinal study had been selected on the basis of a theoretical model that will be outlined with the methods below.

Three main questions were addressed in the analyses. First, the continuity of PSU across time was studied. The second series of cross-sectional analyses compared psychosocial correlates in the PSU groups at each time with the rest of the sample representing the controls. The third question asked for preceding psychosocial markers during middle and late adolescence of later PSU in young adulthood and was, thus, using the longitudinal data.

## Method

### Subjects

Originally, the present sample is based on a cohort of N = 1,964 pupils aged 6 to 17 who were living in the Canton of Zurich, Switzerland in 1994. The cohort was a stratified randomized sample representing the 12 counties of the canton, the school grades, and the types of school and formed the basis of the Zurich Epidemiological Study of Child and Adolescent Psychopathology (ZESCAP). A full description of details of the sampling procedure was given in a previous article [22]. The preadolescents and adolescents (aged 11 – 17 years) of the ZESCAP sample (N = 1110) provided the basic cohort of the longitudinal Zurich Adolescent Psychology and Psychopathology Study (ZAPPS).

This cohort of 1110 subjects was studied longitudinally at three times, namely, in 1994 (time 1), 1997 (time 2) and 2001 (time 3). At each time, a multidimensional screening based on various questionnaires was performed. Subsequently, structured psychiatric interviews were used with those subjects who scored above the cut-off scores and with a certain number of controls scoring below the cut-off score on each screening instrument. Due to the screening procedure the sample was reduced at each time. In addition, subjects dropped out from the sample (e.g. after leaving school) on both the screening and the interview level. At the interview stage compared to the screening stage, a larger proportion of subjects and, particularly, more males than females dropped out. In order to work with a full data set including all questionnaires and interviews based on a sample that still was representative for local census data, the final longitudinal cohort with three waves of assessment was reduced to N = 593. Mean ages of this longitudinal cohort at the three times of assessment were 13.6 (SD = 1.6), 16.6 (SD = 1.6), and 20.2 (SD = 1.7) years. The sample was composed of 284 (47.9%) males and 309 (52.1%) females. These 593 subjects were representative for the census population with regard to gender (Chi^2 ^= 2.14, df = 1, p = n.s.) and biannual age distribution of 17 – 22 years olds (Chi^2 ^= 2.67, df = 2, p = n. s.).

Subjects with problematic substance use in this sample had to fulfil the following criteria: weekly or daily consumption of tobacco, daily alcohol consumption or heavy or problem drinking according to the definition that is given below in the description of the substance use questionnaire, and three or more times use of cannabis during the last month. At time 1, a total of N = 30 subjects were identified who met at least one of these criteria. There were 13 (43%) males and 17 (57%) females in this sub-sample. At time 2, based on the same criteria a total of N = 155 participants were identified who were considered to be subjects. There were 79 (50.1%) males and 76 (49.9%) females in this sub-sample. At time 3, a third PSU group based on the same criteria was defined. This sub-sample comprised a total of N = 290 subjects including 160 (55.2%) males and 130 (44.8%) females. At each time, the rest of the cohort without problematic substance use served as control group. The PSU group was significantly older than the controls at time 1 (15.1 vs.13.5, t = 7.99, df = 36.9, p < .001) and time 2 (17.0 vs.16.4, t = 4.32, df = 299, p < .001) but not at time 3 (20.2 vs. 20.2, df = 591, p = n.s.).

### Measures

The ZAPPS is based on a theoretical model in order to study conditions and processes that are essential to the mental health of growing young people as well as to the development of mental problems and disorders. A broadband questionnaire was chosen in order to obtain information on relevant behavioural and emotional problems of adolescents. Furthermore, various questionnaires dealing with depression, abnormal eating behaviour, and substance abuse were also included. In order to analyze potential risk, compensatory, vulnerability, and protective factors of psychopathology [23], life events were hypothetically seen as stressors, and various psychosocial variables including coping, self-related cognitions, and features of the social network were regarded as moderating factors with regard to behavioural and emotional problems. Questionnaires were filled out confidentially by the subjects during school hours in 1994 and had to be mailed in 1997 and 2001. All questionnaires reflect raw scores and are positively keyed, i.e. high scores represent high expression of the content of the scale.

#### Substance Use Questionaire (SUQ)

The questionnaire was designed by Müller and Abnet [24] in collaboration with the World Health Organization for a nationwide Swiss survey. It covers 22 items that deal both with the consumption of legal drugs and illegal drugs. Nicotine use of both the respondent and his parents is covered by five items. A further eight items deal with alcohol use by the respondent. The response format varied for the different items. The introductory question for nicotine use inquired whether or not the respondent had ever smoked at least one cigarette. For frequency of current nicotine use the scale ranged from "does not apply = 0" to "daily = 3". Alcohol use was assessed via a general introductory question (0 = no consumption, 1 = only a sip, 2 = an entire glass or more) and a detailed list of various alcoholic beverages with a response format ranging from 0 (no consumption) to 5 (daily consumption). Various subgroups were identified and a typology of adolescent alcohol use was validated [21] Two types are relevant for the present study. Heavy drinkers were defined by two positive responses to the following items: I drink until I feel high/until I get drunk. Problem drinkers had to respond positively to the following two items: I drink when I feel lonely/when I feel bad and having a problem.

#### Youth Self Report (YSR)

The problem behaviour section of the YSR [25] and its Swiss adaptation [26] consists of the following primary subscales: socially withdrawn, somatic complaints, anxious/depressed, social problems, thought problems, attention problems, delinquent behaviour, and aggressive behaviour. Two second-order scales reflecting internalizing and externalizing can be calculated. Only these two dimensions were considered in the present analyses. Alpha coefficients of internal consistency for the two scales at the two times ranged from .81 to .87.

#### Young Adult Self Report (YASR)

With the exception of the subscale measuring social problems and the inclusion of the subscale measuring intrusiveness the YASR [27] consists of the same primary and secondary dimension as the YSR [28]. The YASR was used at time 3 (2001) and only the internalizing and externalizing problem scores were considered in the present analyses. The Alpha coefficients amounted to .89 and .80, respectively.

#### Life Event Scale (LES)

A total of 36 items were chosen from pre-existing questionnaires on life events. The time frame was defined as the twelve months prior to filling out the questionnaire. Beside frequencies of life events, a total impact score was calculated. This was based on a scale attached to each item ranging from -2 to +2 and indicating how unpleasant or pleasant the respective event was [29]. The alpha coefficients of internal consistency for the total number of life events ranged from .71 to .73 and for the total impact score from 0.71 to 0.84.

#### Coping Capacities (CC)

Our modified version of the German Coping Across Situations Questionnaire [30] addresses coping in four problem areas with school, parents, peers, and the opposite sex. Factor analysis resulted in two scales measuring active coping and avoidant behaviour. The CC was used at times 1 and 2 and the alpha coefficients of internal consistency for the two scales ranged from .56 to .70.

#### Self-Related Cognitions (SRC)

The ten-item scale for the measurement of self-esteem by Rosenberg [31] and items from a German questionnaire assessing self-awareness [32] were further included into the questionnaire. The latter scale assesses introspective capacities for one's feelings, actions, and past. Alpha coefficients for the two scales across the three assessments ranged from 0.77 to 0.89. The SRC was used at all three times.

#### Perceived Parental Behaviour (PPB)

Based on pre-existing literature, we developed an inventory that consisted of 32 items [33]. Factor analysis resulted in 3 factors explaining 34% of the variance for mothers and 35% of the variance for the fathers. The 3 scales were labelled "acceptance" (e. g., "my mother/father praises me when I do something good"), "rejection" (e. g. "my mother/father easily becomes upset if I don't do what she/he says") and "control" (e. g. "my mother/father has clear rules for my behaviour"). These scales were used only at time 1 and time 2. Alpha coefficients of internal consistency ranged between 0.68 and 0.89.

#### Perceived School Environment (PSES)

These scales were derived from a German project on development in adolescence [34] and consist of 32 items that deal with the perceived psychosocial qualities of the school environment. Our own factorial analyses re-identified the 5 factors labelled "competition among students" (e. g. "in our class, each student tries to be more successful than the other"), "control by the teacher" (e. g. "many of our teachers treat us like small children"), "performance stress" (e. g. "we hardly manage our homework"), "possibility to participate " (e. g. "our teachers ask for our opinion before deciding"), and "peer acceptance" (e. g. "I consider myself to be one of the most accepted students in our class"). These scales were used only at time 1 and time 2. The resulting scales had Alpha coefficients of between .65 and .79 at the two times of assessment.

#### Social Network (SN)

These newly developed scales cover six situations in which emotional or instrumental support is required. For each situation, the questionnaire asks whether or not 9 close individuals (family members, relatives, friends, and teachers) provide support. In addition, the efficiency of each of these individuals is also rated. Factor analyses across situations revealed 2 stable dimensions, namely size and efficiency of the social network with alpha coefficients ranging from .70 to .87 across the three times of assessment.

### Statistical analyses

All questionnaire scores represent raw scores. Data were analysed by use of the 14th version of the SPSS (2006) program. Continuity of substance abuse at risk groups was tested by use of the McNemar Test. Comparisons between risk groups and controls were based on univariate and multivariate analyses of covariance (ANCOVA and MANCOVA) with sex and age as the controlled covariates. Logistic regression analyses were performed stepwise with forward selection in order to identify those variables that allowed the best prediction of cross-sectional and longitudinal risk status.

## Results

The size of the groups with PSU steadily increased from 5.1% risk subjects at time 1 to 26.1% subjects at time 2 and 48.9% subjects at time 3. There was significant continuity from time1 to time 2 with 25/30 (83%) of persistent subjects with PSU compared to 130/563 (23%) of subjects who newly developed PSU (McNemar Test p < .001). In the same way, the PSU group at time 1 showed significantly more PSU also at time 3 (28/30 subjects; 93%) than the control group from time 1 (262/563 subjects; 47%; McNemar Test p < .001). Continuity from time 2 to time 3 was also highly significant with 131/155 (85%) of the subjects with previous PSU showing ongoing PSU whereas only 159/438 (36%) developed PSU de novo (McNemar p < .001). There were also significant gender effects. At time 1, boys were less frequent than girls in the PSU group (13/30 vs. 17/30) and the controls (271/563 vs. 292/563) (McNemar p < .001) whereas males were more frequent among the adolescents at time 2 in the PSU group (79/155 vs. 76/155) and less frequent in the control group (205/437 vs. 233/437) (McNemar p < .001). At time 3 there were no significant gender effects. Furthermore, the PSU group was significantly older than the controls at time 1 (Mean = 15.1, SD = 1.0 vs. Mean = 13.5, SD = 1.6, t = -7.99, df = 36.9, p < .001) and also at time 2 (Mean = 17.0, SD = 1.5 vs. Mean = 16.4, SD = 1.6, t = -4.32, df = 299, p < .001). There were no significant age differences between the two groups at time 3.

In the second step, a series of cross-sectional comparisons between each risk group and the controls was made at each time. Besides significant group effects, there were a large number of significant sex and age effects including interactions of these variables with group. A full presentation of these findings is beyond the scope of this paper. Thus, the presentation will be restricted to group differences based on analyses that took sex and age as covariates into account. Table 1 shows the comparison of emotional and behavioural problems at all three times. As can be seen, the PSU group had highly significantly higher scores for externalizing behaviour at each time whereas there were significant differences for internalizing behaviour only at time 2.

**Table 1:** Comparisons of Emotional and Behavioural Problems (raw scores) at Time 1, Time 2, and Time 3 (N = 593)

	Problematic Substance Use Group		Control Group		
	Total		Total		F
YSR/YASR Secondary Scales	Mean	SD	Mean	SD	(df = 1)
*Time 1*	(N = 30)		(N = 563)		
Internalizing	10.82	6.34	9.05	6.27	2.47
Externalizing	15.24	5.96	9.58	5.73	32.13***
					
*Time 2*	(N = 155)		(N = 438)		
Internalizing	11.40	7.91	9.89	7.24	4.82*
Externalizing	13.68	6.23	8.84	5.12	99.59***
					
*Time 3*	(N = 290)		(N = 303)		
Internalizing	8.59	6.55	8.39	6.72	0.23
Externalizing	8.52	5.60	5.23	3.87	61.46***

*p < .05 **p < .01 ***p < .001

A comparison of the two groups at time 1 with regard to further psychosocial variables is shown in table 2. The PSU group had significantly more life events including a more negative life event impact, and perceived less maternal acceptance, more maternal rejection, more paternal rejection, and more controlling teachers. The corresponding findings for time 2 are shown in table 3. Again, the PSU group experienced a higher number of life events and more negative life events impact, more use of avoidant coping, less parental acceptance and more parental rejection, more controlling teachers, and fewer possibilities to participate at school, but felt more accepted among peers. The pattern of a higher number of life events including a more negative impact of these life events in the PSU group was also seen at time 3 as documented in table 4.

**Table 2:** Comparisons of Psychosocial Correlates of Risk Group and Control Group at Time 1 in 1994

	Problematic Substance Use Group		Control Group		
	Total (N = 30)		Total (N = 563)		F
	Mean	SD	Mean	SD	(df = 1)
Number of Live Events	7.27	3.69	4.36	2.97	19.22***
Life Events Impact	-8.21	5.32	-4.75	4.34	14.60***
Active Coping	5.06	1.37	4.90	2.20	0.00
Avoidant Coping	2.82	1.21	2.71	2.22	0.79
Self-Esteem	25.78	5.33	26.84	5.71	1.64
Self-Awareness	21.70	5.93	18.70	6.69	3.74
Maternal Acceptance	25.00	6.28	28.40	5.59	9.81**
Maternal Rejection	8.50	5.19	6.61	4.38	8.74**
Maternal Control	12.00	3.37	11.23	3.51	2.75
Paternal Acceptance	26.92	18.03	26.64	7.12	0.04
Paternal Rejection	9.41	14.10	6.66	5.27	6.13*
Paternal Control	10.18	9.16	10.31	4.00	0.00
Competition at School	1.44	0.65	1.18	0.73	4.78*
Controlling Teachers	1.99	0.60	1.58	0.72	11.80**
Possibilities to Participate	2.50	0.75	2.66	0.74	2.74
Performance Stress	1.67	0.84	1.24	0.78	3.48
Peer Acceptance	2.92	0.58	2.79	0.71	0.20
Size of Social Network	21.09	5.57	20.60	6.09	0.13
Efficiency of Social Network	21.90	3.30	22.63	3.17	0.37

Group effect: Wilks Lambda = 0.91; F = 3.12; df = 19/571; p < 0.001

*p < .05 **p < .01 ***p < .001

**Table 3:** Comparisons of Psychosocial Correlates of Risk Group and Control Group at Time 2 in 1997

	Problematic Substance Use Group		Control Group		
	Total (N = 155)		Total (N = 438)		F
	Mean	SD	Mean	SD	(df = 1)
Number of Live Events	6.78	3.58	5.00	3.15	31.20***
Life Events Impact	-7.42	5.26	-5.57	4.44	18.08***
Active Coping	4.74	1.23	4.92	1.87	3.60
Avoidant Coping	2.98	1.24	2.60	1.46	11.67**
Self-Esteem	26.69	6.24	27.65	6.00	3.64
Self-Awareness	20.72	5.33	19.94	5.56	1.70
Maternal Acceptance	25.93	6.28	28.11	5.66	15.98***
Maternal Rejection	6.46	4.45	5.40	4.30	10.32**
Maternal Control	9.60	3.94	9.99	3.86	0.25
Paternal Acceptance	23.64	7.04	25.69	6.44	9.83**
Paternal Rejection	6.45	4.62	5.45	3.96	9.07**
Paternal Control	8.47	3.83	9.06	3.78	0.11
Competition at School	8.17	4.64	7.91	4.82	0.46
Controlling Teachers	14.72	6.33	13.39	5.81	11.06**
Possibilities to Participate	14.11	4.47	16.14	4.04	23.85***
Performance Stress	8.75	3.61	8.09	3.87	2.24
Peer Acceptance	15.68	3.03	14.60	3.40	10.53**
Size of Social Network	20.97	6.71	21.31	6.00	0.00
Efficiency of Social Network	21.17	3.13	21.80	3.23	2.90

Group effect: Wilks Lambda = 0.85; F = 5.13; df = 19/571; p < 0.001

*p < .05 **p < .01 ***p < .001

**Table 4:** Comparisons of Psychosocial Correlates of Risk Group and Control Group at Time 3 in 2001

	Problematic Substance Use Group		Control Group		
	Total (N = 290)		Total (N = 303)		F
	Mean	SD	Mean	SD	(df = 1)
Number of Live Events	5.82	3.58	4.42	3.08	26.14***
Life Events Impact	-6.67	5.36	-5.27	4.53	14.04***
Active Coping	5.39	0.99	5.39	1.01	0.38
Avoidant Coping	2.74	1.07	2.60	1.12	1.98
Self-Esteem	26.03	5.33	26.40	5.25	1.36
Self-Awareness	18.94	5.03	18.81	4.93	1.16
Size of Social Network	21.53	6.70	22.65	7.26	2.01
Efficiency of Social Network	22.38	3.46	22.72	3.31	0.5

Group effect: Wilks Lambda = 0.95; F = 4.08; df = 8/582; p < 0.001

*p < .05 **p < .01 ***p < .001

Following these multivariate comparisons of PSU groups and controls, a series of cross-sectional logistic regression analyses was performed in order to identify the strongest predictors of substance use at risk at each time. Findings are shown in table 5. At both times during adolescence, higher age and externalizing problems were strong predictors of PSU. During early adolescence at time 1 there was a strong contribution coming from negative parenting variables to the regression equation, whereas in later adolescence at time 2 it was avoidant coping, perceived lacking possibilities to participate at school, and peer acceptance that contributed to the prediction of PSU. In young adulthood at time 3, only behavioural and emotional problems as reflected by low scores of internalizing and high scores of externalizing problems were predictive of PSU. The overall percentage of correct classification declined from each time to the next time of assessment.

**Table 5:** Significant Predictors of Problematic Substance Use Groups at three times (n = 593)

	B	Wald	p (df = 1)	OR	Nagelkerke R Square	Overall Percentage
**Time 1 Model Summary**					0.449	95.3
Age	0.964	20.776	0.000	2.62		
Externalizing	0.214	15.232	0.000	1.24		
Maternal Acceptance	-0.160	8.534	0.003	0.85		
Maternal Rejection	-0.173	3.952	0.047	0.84		
Maternal Control	0.214	3.922	0.048	1.24		
Parental Rejection	0.139	4.270	0.039	1.15		
Paternal Control	-0.297	8.523	0.004	0.74		
						
**Time 2 Model Summary**					0.341	79.4
Age	0.347	16.725	0.000	1.42		
Externalizing	0.164	41.792	0.000	1.18		
Avoidant Coping	0.219	7.023	0.008	1.25		
Possibilities to Participate	-0.087	7.812	0.005	0.917		
Peer Acceptance	0.146	9.514	0.002	1.16		
						
**Time 3 Model Summary**					0.198	64.6
Internalizing	-0.062	7.995	0.005	0.940		
Externalizing	0.164	40.347	0.000	1.18		

Longitudinal analyses looked for time 1 and time 2 predictors of PSU at time 3. Results may be seen in table 6. Amongst the various variables that were assessed in early adolescence at time 1, PSU, low scores on the internalizing dimension, high score on the externalizing dimension, self-awareness, a lack of perceived possibilities to participate at school, and male gender allowed a significant prediction of PSU at time 3 in young adulthood. At time 2 in late adolescence the significant predictors were PSU again that was supplemented by externalizing problems, perceived paternal acceptance, and male gender. When data from both times in adolescence were used for prediction the following variables allowed a significant prediction of PSU at time 3: PSU both at time 1 and 2, low scores on the internalizing dimension at time 1, externalizing problems at time 1, self-awareness at time 1, perceived paternal acceptance at time 2, and male gender.

**Table 6:** Significant Predictors of Problematic Substance Use (PSU) at Time 3 by Time 1 and Time 2 Variables

	B	Wald	p (df = 1)	OR	Nagelkerke R Square	Overall Percentage
**Time 1 Model Summary**					0.251	66.8
PSU at time 1	2.304	8.841	0.003	0.10		
Internalizing	-0.066	10.078	0.002	0.94		
Externalizing	0.094	19.097	0.000	1.10		
Self-Awareness	0.050	8.332	0.004	1.05		
Possibilities to Participate	-0.370	7.263	0.007	0.69		
Male Gender	0.588	8.625	0.003	1.80		
						
**Time 2 Model Summary**					0.335	72.2
PSU at time 2	2.078	57.335	0.000	0.13		
Externalizing	0.052	5.017	0.025	1.05		
Paternal Acceptance	0.055	5.336	0.021	1.06		
Male Gender	0.620	8.126	0.004	1.86		
						
**Time 1 and 2 Model Summary**					0.403	72.7
PSU at time 1	1.870	5.295	0.021	0.15		
PSU at time 2	1.894	40.623	0.000	0.15		
Internalizing at time 1	-0.053	4.207	0.040	0.95		
Externalizing at time 1	0.055	4.653	0.031	1.06		

## Discussion

The present study compared PSU groups and control groups in the community rather than clearly defined clinical groups fulfilling criteria for manifest substance abuse. The emphasis on PSU was taken because these subjects should be potentially more apt and open for preventive measures. If there are cheap, reliable, and valid assessment tools for the identification of PSU subjects, identification in the community would be the first step in order to start interventions. These tools should not only be simple to administer but also allow composite definitions of substance use. Rather than focussing only on a single substance the present study is based on a composite definition of PSU reflecting the fact that most adolescent and young adult users consume various substances. Furthermore, the present longitudinal study allowed both for the repeated analysis of cross-sectional correlates and the predictive power of these variables on substance use at risk.

Starting from the identification of PSU by a combination of items asking for the frequency of alcohol, tobacco, and cannabis use, a steadily increasing number of subjects across the time span from early adolescence to young adulthood was identified in the present study. A more than nine-fold increase of PSU was observed in this period with a slight majority of females in a rather small risk group in early adolescence and a male predominance in males at later times. The increasing number and significant continuity of PSU is very much in line with what can be expected from recent reviews of international trends in use of a range of substances [13,20].

In the next step of the analyses it was shown that PSU at each point in time of development was significantly associated with a number of behavioural and other psychosocial features. These correlates were not only important by themselves but also served as a validation of the definition of PSU. There was a clear association with externalizing problems across all three times which is also very much in accordance with other studies on developmental patterns of substance use and abuse [1,5,7-9,13,18-20]. However, at time 2 in late adolescence PSU subjects also showed higher scores for internalizing problems. This finding matches other studies that found mood and anxiety disorders to be frequently associated with substance use disorders in adolescents [20].

Among the various psychosocial correlates the most consistent finding of PSU was the persistent association with life events from early adolescence to young adulthood which is in line with the finding that negative life events contribute to escalated substance use during adolescence [6]. Also in line with other studies [6,17] is the consistent finding that low parental support in terms of lower perceived parental acceptance and higher rejection at both times during adolescence is a marker of PSU. Avoidant coping as an additional correlate of substance use at risk was identified only at time 2 in late adolescence, which has been observed in terms of non-adaptive coping as another association with escalating substance use in adolescence [6]. Finally, among the various correlates of perceived school environment during adolescence, PSU was most consistently characterized across two times by the more controlling behaviour of the teacher, and in late adolescence also by the lack of possibility to participate and a higher amount of perceived peer acceptance.

The joint consideration of the correlated variables in logistic regression analyses allowed a more definite evaluation of the most relevant associations with PSU. At times 1 and 2 during adolescence, age was strongly contributing to the prediction of risk status indicating that there is a developmental pattern with increasing age contributing to PSU. The second strongest and most persistent variable across all three times contributing to PSU are externalizing problems. At younger age in early adolescence, negative perceived parenting contributes significantly to PSU whereas with greater autonomy in late adolescence these associations are replaced by deficits in active problem solving as indicated by avoidant coping, a feeling of having a lack of possibilities to participate at school, and peer influences as reflected by high peer acceptance. Later in young adulthood, none of the psychosocial variables besides externalizing problems (and the reverse, i.e. low scores on the internalizing dimension) had any predictive power. However, it should be taken into account that there were less potential correlates assessed at this time compared to the two previous times in the study.

The longitudinal analyses showed that at each time during adolescence it is predominantly the preceding PSU status that predicts PSU at outcome in young adulthood. Thus, the self-perpetuating character and the high rate of persisting PSU were underlined again by the data in this study. Furthermore, age was a predictor only at time 1 and 2. Together with the increased age of the PSU groups at these times, this finding points to the rather trivial fact that PSU is age-dependent during adolescence. Other major and persistent risk factors were externalizing problems and male gender. Despite the trend for a closing gender gap with increasing age in a previous cross-sectional analysis of substance use in an even larger sample [16], the present approach with a focus on specifically defined subjects emphasizes the particular risk of males for remaining PSU subjects across time. Among the other psychosocial variables only a few contributed to the prediction and included some variables that were assessed in early adolescence, namely, low scores on the internalizing dimension which is just the correlate of the externalizing problems, heightened self-awareness, and a feeling of lacking possibilities to participate at school. In late adolescence, only perceived paternal acceptance contributed to the prediction which may be due to the fact that the father was seen as a role model particularly for the males in the PSU group.

In conclusion, the present study points to a rather persistent pattern of problematic substance use with a number of associated and predictive psychosocial features that both can be assessed in the community as a first step for the identification of individuals who are in danger of developing long-term risk behaviours in adulthood. The strength of the present study lies in the large community sample and the longitudinal approach. Limitations include the emphasis on PSU, rather than, substance use disorders according to the major schemes of diagnostic classification, and the reliance on self-reports of the subjects. However, it should be kept in mind that at this age in adolescence and early adulthood parents and caretakers are less reliable informants as to substance use in youths.
