# Continuity, psychosocial correlates, and outcome of problematic substance use from adolescence to young adulthood in a community sample

**DOI:** 10.1186/1753-2000-3-37

**Published:** 2009-11-23

**Authors:** Hans-Christoph Steinhausen, Susanne Eschmann, Christa Winkler Metzke

**Affiliations:** 1 Department of Child and Adolescent Psychiatry, University of Zurich, Neumuensterallee 9, CH 8032 Zurich, Switzerland

## Correction

Following publication of this work [1] we found there were some errors in line 6-9 of the results section. In the published version we stated:

At time 1, boys were less frequent than girls in the PSU group (13/30 vs. 17/30) and the controls (271/563 vs. 292/563) (McNemar p < .001) whereas males were more frequent among the adolescents at time 2 in the PSU group (79/155 vs. 76/155) and less frequent in the control group (205/437 vs. 233/437) (McNemar p < .001). At time 3 there were no significant gender effects.

The correct text should read as follows:

At time 1 and 2, there were no significant gender effects in the PSU groups (t1: 13/30 vs. 17/30; t2: 79/155 vs. 76/155; Chi2 = 0.26, df = 1, p = 0.61) and among the controls (t1: 271/563 vs. 292/563; t2: 205/438 vs. 233/438; Chi2 = 0.80, df = 1, p = 0.37). In contrast, at time 3 there were more males than females in the PSU group (160/290 vs. 130/290) and less males than females in the control group (124/303 vs. 179/303) (Chi2 = 12.05, df = 1, p < 0.001).
